# CAR T cell therapy in autoantibody-mediated neurological disorders: a promising strategy

**DOI:** 10.1186/s12974-025-03662-6

**Published:** 2026-03-12

**Authors:** Muzi Wen, Ruoyi Zheng, Hanqing Zhang, Sophia Y. Goldberg, Zhiying Jian, Ye Gao, Ruogu Cheng, Linxin Wen, Yu Zhao, Saad S. Kenderian, Pei Shang

**Affiliations:** 1 Department of Neurology, Nanfang Hospital, Southern Medical University, Guangzhou Road North 1838#, Guangzhou, 510515 China; 2 The First School of Clinical Medicine, Nanfang Hospital, Southern Medical University, Guangzhou, China; 3 Department of Neurology, Mayo Clinic, Rochester, MN USA; 4 College of Biological Sciences, University of California, Davis, USA; 5 T Cell Engineering Program, Mayo Clinic, Rochester, MN USA; 6 Mayo Clinic Medical Scientist Training Program, Mayo Clinic, Rochester, MN USA; 7 Department of Immunology, Mayo Clinic Graduate School of Biomedical Sciences, Rochester, MN USA; 8 Division of Gastroenterology and Hepatology, Mayo Clinic, Rochester, MN USA; 9 Department of Hematology, Mayo Clinic, Rochester, MN USA; 10 The Second School of Clinical Medicine, Nanfang Hospital, Southern Medical University, Guangzhou, China

**Keywords:** Autoantibody-mediated neurological disorders, Anti-NMDA receptor encephalitis, CAR T cell therapy, Diacylglycerol lipase alpha antibody associated encephalitis, Neuromyelitis optica spectrum disorder, MOG antibody-associated disease, Myasthenia gravis, Lambert-Eaton myasthenic syndrome, Multiple sclerosis, Stiff person syndrome

## Abstract

**Background:**

Chimeric antigen receptor (CAR) T cell therapy is emerging as a promising approach for B cell-driven neurological autoimmune disorders, particularly those characterized by pathogenic autoantibodies that target key neural structures. These conditions, including neuromyelitis optica spectrum disorder, myasthenia gravis, Lambert-Eaton myasthenic syndrome, MOG antibody-associated disease, anti-NMDA receptor encephalitis, Diacylglycerol lipase alpha antibody associated encephalitis, stiff person syndrome, and multiple sclerosis, can be categorized based on their primary autoantigens into (1) extracellular antigen-associated (e.g., AQP4, AChR, NMDAR, MOG, VGCC), (2) intracellular antigen-associated (e.g., GAD65, DAGLA), or (3) unidentified antigenic origin (as seen in multiple sclerosis). This distinction is essential for guiding therapeutic strategies and exploring novel principles represented in distinct treatment approaches and their corresponding therapeutic outcomes.

**Main body:**

In this review, we propose a classification of CAR T cell therapies designed for different target antigens, including: CD19/20/BCMA-directed CAR T cells targeting general B cell-mediated pathogenesis, regulatory T cells modified with CARs, and the design of chimeric autoantibody receptors (CAARs) to selectively deplete pathogenic B cells directly associated with disease progression while preserving immune tolerance. We further discuss preclinical and clinical advancements, key challenges such as safety concerns and neurotoxicity, and the future landscape of CAR T applications in neuromyelitis optica spectrum disorder, myasthenia gravis, Lambert-Eaton myasthenic syndrome, MOG antibody-associated disease, anti-NMDA receptor encephalitis, Diacylglycerol lipase alpha antibody associated encephalitis, stiff person syndrome, and multiple sclerosis according to the latest research, case and trial data.

**Conclusion:**

CAR T cell therapy potentially offers a highly specific and effective method with thorough elimination of autoreactive B cells, representing a rapidly evolving field with the potential to transform the treatment of autoimmune neurological disorders. As CAR T technology advances, it holds the potential to become a groundbreaking immunoablative strategy with so-far disclosed controllable side effects; however, further long-term follow-up data are still needed to validate its application in autoimmune disorders.

## Background

Autoimmune disease is a pathological process in which the body’s immune tolerance is disrupted, leading to a sustained pathological immune response to autoantigens and causing damage to normal cells or tissues, accompanied by the development of autoantibodies or autoreactive T cells [1]. In recent years, significant progress has been made in developing novel therapies for autoimmune diseases. Among these, autoimmune diseases that affect the nervous system present unique challenges due to their complex mechanisms, the distinctive anatomical locations, and the restrictive nature of the blood-brain barrier (BBB), which impedes the action of many therapeutic agents. Although an increasing number of innovative treatments have been developed with relapse rate significantly improved, there remains a substantial proportion of individuals with neuroimmunological disorders, such as myasthenia gravis (MG) or neuromyelitis optica spectrum disorders (NMOSD), that fail to achieve the expected disease improvement under current therapies [2, 3]. This highlights the existence of a distinct subgroup of refractory clinical cases. Yet, the safety profile of antibody-based therapies for this group remains a matter of ongoing debate [4].

Humoral immunity-mediated autoimmune diseases associated with the nervous system include MG, NMOSD, multiple sclerosis (MS), anti-N-methyl-D-aspartate acid receptor (Anti-NMDAR) encephalitis, stiff-person syndrome (SPS), and myelin oligodendrocyte glycoprotein antibody-associated disease (MOGAD). The roles of self-reactive B cells and the autoantibodies produced in the progression of neuroimmunological diseases have been further elucidated. Specific mechanisms include: (1) generating antibodies that injure tissue by activating complement or antibody-dependent cell-mediated cytotoxicity [5]; (2) stimulating antigen-presenting cells (APCs), which activate self-reactive T cells and promote the production of cytokines [6]; (3) releasing pro-inflammatory cytokines that activate macrophages, thereby exacerbating tissue damage [7]; (4) blocking cell surface receptors (e.g., MG) [8]; (5) upregulating cell surface receptors (e.g., Graves’ disease) [9]; (6) disrupting cell adhesion (e.g. pemphigus vulgaris (PV)) [10]; (7) cell surface receptors internalization or downregulation (e.g., Anti-NMDAR encephalitis). These mechanisms primarily function through extracellular or membrane-bound targets. Consequently, for neurological autoimmune diseases with pathogenic antigens located extracellularly or on the cell membrane, such as NMOSD and Anti-NMDAR encephalitis, B cell depletion therapies have been validated by retrospective data and show robust clinical efficacy [11, 12].

However, due to inherent limitations of traditional B-cell depletion therapies, such as incomplete elimination of pathogenic B-cell subsets and limited durability of response, the exploration of more effective approaches has gained momentum, particularly Chimeric Antigen Receptor (CAR) T cell therapy. CAR T cells have the potential to penetrate deeper tissues and are not restricted by major histocompatibility complex (MHC) binding [13, 14]. The limited effectiveness of monoclonal antibody therapies in neurological autoimmune diseases such as NMOSD, MS, and Anti-NMDAR encephalitis is partly attributable to their inability to fully eliminate all pathogenic B cell subsets, especially long-lived plasma cells and memory B cells residing in secondary lymphoid tissues, which may escape CD20-based depletion and contribute to residual autoantibody production or relapse [15, 16]. Meanwhile, CAR T cell therapy may allow for more efficient and thorough depletion of autoreactive B cells, thus forcing a complete reset of the immune system, even in patients who do not properly respond to B cell depletion therapy strategies [17].

Through its potential for precise and sustained B-cell elimination, CAR T cell therapy represents an innovative strategy that can complement and, in certain aspects, extend the benefits achieved with monoclonal antibody–mediated B-cell depletion. Several clinical trials in systemic autoimmune diseases have already demonstrated its promise [18, 19]. For instance, in the case of systemic lupus erythematosus (SLE), CD19 CAR T cell therapy has been observed to induce a significant and rapid decline of dsDNA antibodies, suggesting that CAR T cell therapy can induce profound depletion of autoreactive B cells in tissues, accompanied by only a slight decrease in total immunoglobulin levels [20]. Moreover, long-term clinical follow-ups in various autoimmune diseases, such as SLE, systemic sclerosis (SS), and idiopathic inflammatory myopathies (IIM), have revealed that the side effects associated with CD19-targeted CAR T cell therapy are less severe than expected and remain manageable [21]. This reduction in symptoms is likely due to the considerably lower number of pathogenic B cells in autoimmune diseases than B cell-derived malignancies [22, 23]. This can potentially reduce the risk of toxic reactions such as cytokine release syndrome (CRS) and immune effector cell-associated neurotoxicity syndrome (ICANS) linked to CAR T cell therapy.

Meanwhile, experimental results indicate a substantial reduction or even complete disappearance of autoantibodies, accompanied by relatively mild side effects and sufficient retention of immunoglobulin levels [24–30]. Some hypotheses have been proposed to address this paradox of comprehensive B cell depletion, suggesting that CD19 is still expressed on plasmablasts. At the same time, CD19^-^ and CD20^-^ long-lived plasma cells in the bone marrow can continue to produce adequate protective antibodies, which enables CD19-targeted therapy to achieve thorough elimination of pathogenic B cells without compromising the body’s normal immune function [31, 32]. The different roles of short-lived and long-lived plasma cells in SLE have been revealed, with pathogenic anti-dsDNA antibodies predominantly originating from CD19^+^ short-lived plasma cells and plasmablasts [20]. This finding is closely related to their varying sensitivities to B cell-targeted therapies in SLE, and a similar approach holds promise for broader application to other neurological autoimmune diseases [33].

The efficacy of monoclonal antibodies targeting different B cell markers and CAR T cell therapies in depleting B cells can be helpful in deciphering the cellular origins of autoantibodies in various autoimmune diseases. Specifically, these therapies help determine the extent to which autoantibodies are derived from plasmablasts, short-lived, or long-lived plasma cells. Although current study cohorts remain limited in size, the preliminary findings are promising and support the potential safety of CAR T cell therapy for broader clinical application in neurologic autoimmune diseases [34, 35]. Apart from the curative outcomes, the use of CAR T cell therapy has also shown the potential to bring the dysregulated immune system to a halt, which hints at the possibility of complete restoration of immune tolerance [36].

This article summarizes the latest advances of preclinical and clinical trials of CAR T cell therapy in neurological autoimmune diseases based on the classification of autoantibody targets. These targets are classified as extracellular antigen (MG, MOGAD, Lambert-Eaton Myasthenic Syndrome (LEMS), Aquaporin 4 (AQP4)-NMOSD, Anti-NMDAR encephalitis), intracellular antigen (SPS, Diacylglycerol lipase alpha (DAGLA) antibody associated encephalitis) and unspecified orgin (MS). Categories of CAR T cell therapies were further distinguished based on their target specificity, separating approaches directed at general B cell populations from those engineered to recognize specific autoantigens. The applications of CAR T cell in various autoimmune diseases have deepened our understanding of how aberrant B cell activation contributes to the disease pathogenesis.

## Main text 

### Current therapies and their limitations

First-line therapies for autoimmune diseases consist of glucocorticoids, intravenous immunoglobulin (IVIG), and plasma exchange, while second-line therapies include B cell depletion therapy and cyclophosphamide administration. Glucocorticoids play a central role, as they not only suppress inflammatory cascades but also inhibit lymphocyte proliferation and antibody synthesis, thereby directly modulating the autoimmune responses [37]. Immunomodulatory therapies (e.g., plasma exchange and IVIG) and targeted immunosuppressive therapies (e.g., tumor necrosis factor (TNF) inhibitors, B cell depletion therapy, and T cell-targeted therapies) aim to regulate the immune system and promote beneficial immune responses in patients [38].

While biologics and small-molecule therapies have shown potential, they hardly account for the variability of the underlying pathophysiology and individualized medication responses of autoimmune disease. Monoclonal antibody therapy, which targets B cell surface molecules to disrupt aberrant B cell function, has also emerged as an effective immunotherapeutic approach for treating autoimmune diseases. Rituximab, a CD20-targeting monoclonal antibody, has been widely adopted as a second-line immunotherapy for refractory autoimmune encephalitis including Anti-NMDAR encephalitis [39]. Antibodies or agents targeting plasma cells (e.g., the proteasome inhibitor bortezomib), cytokines (e.g., the anti-IL-6 receptor antibody tocilizumab), or complement components (e.g., the anti-C5 antibody eculizumab) have been employed in clinical practice for refractory cases of autoimmune encephalitis and neuroimmunological conditions such as MG and NMOSD [40, 41]. Bortezomib has been reported to reduce pathogenic antibody production and improve outcomes in refractory Anti-NMDAR encephalitis in several case series, while tocilizumab has shown benefit in rituximab-refractory autoimmune encephalitis cohorts [42, 43]. Eculizumab has also gained robust evidence and regulatory approval for AQP4-IgG-positive NMOSD and for certain indications in MG. Besides, CD19-directed B-cell depletion with inebilizumab has been approved for AQP4-IgG-positive NMOSD, and anti-CD20 agents such as ocrelizumab and ofatumumab have gained approval by the FDA and EMA for MS, illustrating the expanding arsenal of B-cell-targeted therapies across neuroimmunological diseases [11, 44].

Despite these advances, clinical practices still reveal certain limitations. For instance, in multiple sclerosis, emerging evidence suggests that while relapses may be temporarily suppressed, disease progression continues unabated after treatment of MS with monoclonal antibodies [45]. Similar challenges exist across neuroimmunological disorders, where pathogenic immune subsets are not completely eradicated, and relapses or residual deficits frequently persist. As seen in NMOSD where, despite substantial reductions in annualized relapse rate by monoclonal antibodies, a non-trivial proportion of patients still experience relapses during treatment with cumulative disability [16]. These observations highlight the need for next-generation strategies, such as cellular therapies of CAR T cells, that may potentially achieve deeper and more durable immune modulation with greater specificity.

### The principles of car t cell therapy

CAR T cell therapy is a novel treatment that harnesses a patient’s immune system. By isolating T cells from the patient’s peripheral blood and modifying them through bioengineering and ex vivo expansion, T cells are redirected to recognize and eliminate target cells, achieving precision therapy specifically. Additionally, CAR T cells’ antigen recognition is not restricted by MHC, allowing them to be designed for various targets and address a broader range of antigens [46]. Currently, CAR T cell therapy mainly targets B cell-related tumor-associated antigens (TAAs), such as CD19, CD20 and BCMA, thereby combining T cell killing efficacy with antibody specificity [47].

**Fig. 1:**
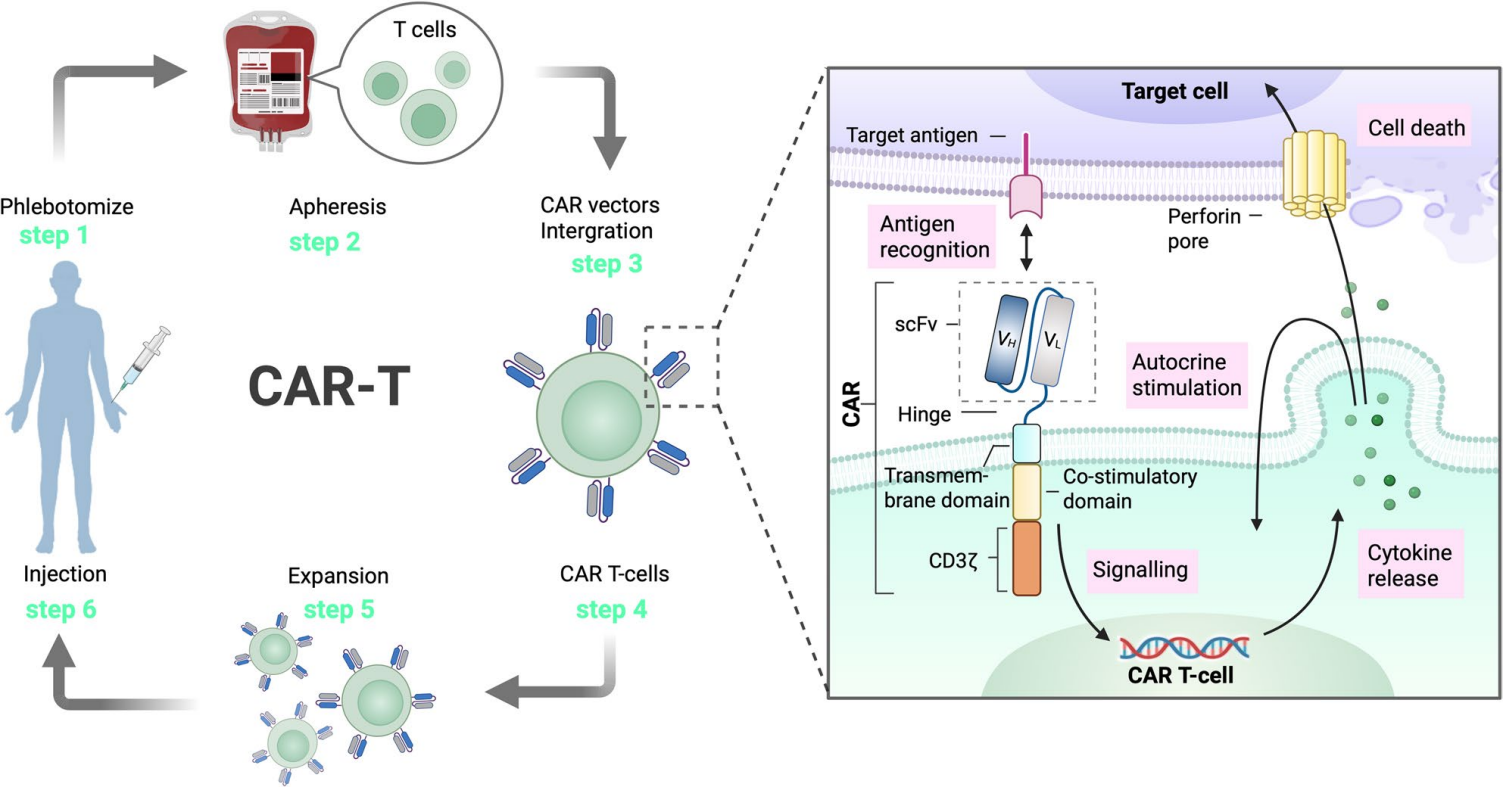
The production and design of CAR T cell therapy process and its mechanism of action Six key steps are involved in CAR T cell production: (1) Phlebotomy, where peripheral blood is collected from the patient; (2) Apheresis, a process to isolate T cells; (3) CAR vector integration, where T cells are genetically modified to express CARs; (4) CAR T cell generation, where the modified T cells express CARs on their surface; (5) Expansion, where CAR T cells undergo *in vitro* proliferation; (6) Injection, where the expanded CAR T cells are infused back into the patient

Structurally, CAR T cell constructs comprise three parts: the extracellular domain, the transmembrane spacer region, and the intracellular domain. The extracellular domain fuses monoclonal antibody single-chain fragment variables (scFv) for antigen specificity followed by a hinge region. ScFv is the foundation for CAR T cell’s ability to specifically bind pathogenic antigens, comprising the light chain (V_L_) and heavy chain (V_H_) of monoclonal antibodies linked via a polypeptide chain. The transmembrane spacer region, often involving the CD3ζ (zeta), CD8, or CD28 chains, connects the extracellular region to the intracellular signaling domain. Meanwhile, the intracellular domain comprises the signaling domain of the T cell receptor (TCR) and co-stimulatory domains, usually derived from the CD28 receptor family or tumor necrosis factor receptor families such as 4-1BB and CD27. These are followed by the CD3ζ stimulatory domain, whose function is to achieve dual activation through co-stimulatory signaling and intracellular pathways, thereby enabling T cells to proliferate continuously, secrete cytokines, and enhance cytotoxic activity. Notably, all FDA-approved commercial CAR T cell products are based on second-generation designs that target CD19 or BCMA, with either CD28 or 4-1BB co-stimulatory domains and are used for treating relapsed or refractory hematologic malignancies. These therapies have shown outstanding therapeutic efficacy against B cell-mediated malignancies [48–50] (Fig. 1).

The mechanism of CAR T cell targeting, and specific killing is attributed to its prototypical CAR construct, consisting of a scFv for antigen recognition, a hinge region, a transmembrane domain, a CD3ζ signaling domain, and a co-stimulatory domain. Upon target antigen recognition, CAR T cells become activated, leading to perforin-mediated cytotoxicity, cytokine release, and autocrine stimulation, ultimately inducing target cell apoptosis.

Remarkably, CAR design has expanded to include other engineered cell populations and additional targets besides the conventional B cell markers. CAR-regulatory T cells (CAR-Tregs) show promise in restoring immune tolerance and controlling autoimmune responses [51]. As Treg cells serve as the brakes to switch off the overreactive immune system, they are ideally suited to address autoimmune conditions. Therefore, engineering Treg cells with CARs enables them to acquire antigen specificity and traffic to areas of inflammation. In addition to CAR T cell therapies targeting broadly expressed B-cell antigens (e.g., CD19, CD20), the development of chimeric autoantigen receptor (CAAR) T cells, which selectively eliminate autoreactive B cells while sparing the non-pathogenic B-cell repertoire, highlights a strategy with the potential to achieve more precise targeting and improved immune tolerance in immune-mediated diseases [52].

In theory, CAR T cell therapy can be effective in diseases where a specific pathogenic cell population exists and can be precisely targeted. Therefore, autoimmune diseases characterized by partial aberrations of B cells that produce autoantibodies perfectly align with CAR T cell therapy’s application model. Particularly for the autoimmune diseases associated with central nervous system (CNS), which is isolated from the rest of the body by the BBB, CAR T cells represent an effective tool for deep penetration into the CNS compartment, due to T cells’ natural infiltration ability, high-affinity specific target binding, and anti-target effector functions. The prior clinical success of CAR T cell therapy has inspired exploration beyond the field of non-solid tumors, with recent advances in solid tumors and autoimmune diseases.

There are two hallmark clinical toxicities that affect patient outcomes and CAR T cell therapy function: CRS and ICANS. CRS arises from a high-level amplification and target cell lysis triggered shortly after CAR T cell binding to its target antigen, which results in a massive release of cytokines into the body [53]. ICANS, on the other hand, has a less well-defined pathogenesis. Some studies suggest it is associated with damage to the BBB, allowing CAR T cells and other immune cells to migrate into the cerebrospinal fluid (CSF), which leads to the release of intermediate-to-high levels of pro-inflammatory cytokines such as interleukin-6 (IL-6) and interleukin-8 (IL-8) [54]. Given the variability of adverse events reported across CAR T cell adoption, we outline the grading criteria used throughout this review (Table 1).

**Table 1:** CAR-T cell therapy related adverse events and grading criteria

Complication Category	Grade	Specific Manifestations
Cytokine Release Syndrome (CRS) (54)	Grade 1	Fever only (≥ 38 °C)
	Grade 2	Hypotension; Hypoxia relieved by low-flow nasal cannula
	Grade 3	Hypotension requiring vasopressors; Hypoxia requiring high-flow nasal cannula
	Grade 4	Hypotension requiring multiple vasopressors; Hypoxia requiring mechanical ventilation
Immune Effector Cell Associated Neurotoxicity Syndrome (ICANS) (54)	Grade 1	ICE score 7–9; CAPD score 1–8; arousable spontaneously; no seizures; no motor impairment; no cerebral edema
	Grade 2	ICE score 3–6; CAPD score 1–8; arousable to voice; seizure responsive to intervention or non-convulsive seizures on EEG; no motor impairment; localized cerebral edema on imaging
	Grade 3	ICE score 0–2; CAPD ≥ 9; arousable only to tactile stimulus; life-threatening seizure ≥ 5 min or repetitive seizures; severe focal motor weakness (e.g., hemiparesis, paraparesis); diffuse cerebral edema involving cranial nerves/ optic disc
	Grade 4	ICE score 0 (unable to assess); CAPD not testable; unarousable/ coma; status epilepticus or uncontrolled electrographic seizures; profound focal motor deficits (e.g., hemiplegia); severe cerebral edema with brainstem involvement or herniation
Infections	/	Bacterial, viral, fungal infections
Other Complications	/	Allergic reactions or anaphylaxis (e.g., persistent skin rash)
Hematological Complications	/	Prolonged cytopenia; B-cell aplasia and hypogammaglobulinemia
Anemia (Hemoglobin, g/dL)	Grade 1	< LLN-10.0
	Grade 2	< 10.0–8.0
	Grade 3	< 8.0; transfusion indicated
	Grade 4	Life-threatening; urgent intervention required
Thrombocytopenia (Platelet count, ×10⁹/L)	Grade 1	< LLN − 75
	Grade 2	< 75 − 50
	Grade 3	< 50 − 25; transfusion indicated
	Grade 4	< 25; life-threatening consequences; urgent intervention
Neutropenia (Absolute neutrophil count, ×10⁹/L)	Grade 1	< LLN-1.5
	Grade 2	< 1.5-1.0
	Grade 3	< 1.0-0.5
	Grade 4	< 0.5; life-threatening; urgent intervention

*ICE score* Immune effector Cell-Associated Encephalopathy score; lower scores indicate more severe neurotoxicity, *CAPD score* Cornell Assessment of Pediatric Delirium score; higher scores indicate more severe delirium, *LLN* Lower limit of normal

Reference for hematologic grading: U.S. Department of Health and Human Services, National Institutes of Health, National Cancer Institute. Common Terminology Criteria for Adverse Events (CTCAE) Version 5.0. Bethesda, MD: National Cancer Institute

(Available at:https://ctep.cancer.gov/protocolDevelopment/electronic_applications/ctc.htm#ctc_50)

Therefore, finding a delicate balance between the side effects of CAR T cells and the management of disease progression in the field of autoimmune diseases strongly appeals for the support of more data and trials. However, the above side effects, in terms of their severity and incidence, are significantly less pronounced in noncancerous contexts, especially in autoimmune diseases, comparing with the challenges faced in the application of CART cell therapy in the field of solid tumors. The target cell population in autoimmune diseases is smaller, with a lower mutational burden and genetic heterogeneity, therefore the severity of side effects, such as CRS, resulting from the clearance of pathogenic B cells is much lower [55]. This has been supported by long-term clinical follow-up in patients with SLE, SSC, and IIM; however, these findings remain limited by small sample sizes [21]. Moreover, the risk of antigen mutation and subsequent immune escape, which represents a major challenge for CAR T cell therapy in oncology due to high antigen load and tumor heterogeneity, is not a concern in autoimmune diseases [56]. In these disorders, the pathogenic autoantigens are relatively stable, eliminating the problem of antigen variability. Particularly with CAAR T cell therapy, the approach achieves selective depletion of autoreactive B cells that express pathogenic autoantibodies, while sparing the protective B cell repertoire. This confers superior specificity and immune tolerance compared with applications in solid tumors, where both antigen escape and off-target cytotoxicity remain significant barriers [29, 57].Thus, the concept of using CAR T cells to target autoimmune disorders is highly compelling and holds great potential for synergizing with endogenous immune functions to eliminate pathological cells. However, further data will be needed to provide a clearer and more convincing understanding moving forward [58].

### Classifications of intracellular and extracellular antigens in neurological autoimmune diseases and car T cell therapy

#### Understanding the classification of intracellular and extracellular autoantibody-mediated neurological disorders

Neural autoantibodies play a vital role in various neurological autoimmune disorders. Therefore, autoantibodies specific to neuronal, glial, or muscle antigens can serve as diagnostic biomarkers or pathogenic factors in neuroimmunological diseases. These neural autoantibodies can be classified based on the location of their target antigens: (1) intracellular proteins such as nuclear proteins, cytoplasmic enzymes, and RNA-binding proteins, including ANNA-1 (Anti-Hu), ANNA-2 (Anti-Ri), ANNA-3, Ma1/Ma2, Purkinje Cell Antigen 1/2(PCA-1/2; Yo/Tr), Amphiphysin, Glutamic Acid Decarboxylase 65 (GAD65), and glial fibrillary acidic protein α (GFAPα); and (2) membrane and synaptic proteins (ion channels, water channels, and neurotransmitter receptors), such as AQP4, γ-aminobutyric acid receptors (GABA-R), MOG, NMDAR, anti-acetylcholine receptor (AChR), α-amino-3-hydroxy-5-methyl-4-isoxazolepropionic acid receptor (AMPAR), Muscle-Specific Kinase (Musk), Voltage-Gated Calcium Channel (VGCC), N-Methyl-D-Aspartate Receptor Subunit 1 (NR1), and Glutamate Receptor 1/2 (GluR1/2) [59, 60] (Table 2).

**Table 2:** Antibody-mediated neurological disorders

Classification	Antibodies	Common Phenotypes
Extracellular Antigens	Muscle AChR	MG
	Neuronal (α3) AChR	Dysautonomia
	Neuronal (α7) AChR	Encephalitis
	MuSK	MG
	VGKC	Reversible autoimmune encephalopathy
	VGCC	LEMS
	AQP 4	NMOSD
	NMDAR	LE, seizures, catatonia, subacute memory disturbance, abnormal movements
	Glycine receptor	SPS, encephalomyelitis, seizures, rigidity, myoclonus,
	MOG	ON, ADEM, myelitis
	LGI1	LE, faciobrachial dystonic seizures, neuromyotonia
	CASPR2	LGI1-like, neuromyotonia and Morvan’s syndrome, neuropathic pain
	GABA_B_ receptor	LE, seizures, memory loss
	GABA_A_ receptor	Encephalitis, seizures, catatonia
	mGluR5	LE, seizures, movement disorders
	Neurexin 3α	Clinical overlap with Anti-NMDAR encephalitis
	Septin 7	Encephalitis
Intracellular Antigens	Striational muscle	MG, myositis
	Amphiphysin	SPS, encephalomyelitis
	Synapsin	LE, neuropsychiatric disorders
	GFAP	Meningoencephalomyelitis
	GAD65	LE, SPS, seizures
	Ma	LE, diencephalic dysfunction, brainstem encephalopathy
	ANNA-1 (Anti-Hu)	LE, sensory neuropathy
	ANNA-2 (Anti-Ri)	Movement disorders, parkinsonism
	AK5	LE
	AGNA (SOX1)	LEMS

*Abbreviations*: *AChR* Nicotinic acetylcholine receptor, *ADEM* Acute disseminated encephalomyelitis, *AGNA* Antiglial nuclear antibody, *AK5* Adenylate kinase 5, *ANNA* Antineuronal nuclear antibody, *AQP4* Aquaporin-4 water channel, *CASPR2* Contactin-associated protein-like 2, *GABA* γ-aminobutyric acid, *GFAP* Glial fibrillary acidic protein, *LE* Limbic encephalitis, *LGI1* Leucine-rich glioma-inactivated 1, *LEMS* Lambert–Eaton myasthenic syndrome, *MG* Myasthenia gravis, *mGluR5* Metabotropic glutamate receptor 5, *MuSK* Muscle-specific tyrosine kinase, *NMDAR* N-methyl-d-aspartate receptor, *NMOSD* Neuromyelitis optical spectrum disorder, *ON* Optic neuritis, *SPS* Stiff person syndrome, *VGCC* Voltage gated calcium channel, *VGKC* Voltage-gated potassium channels

Autoantibodies targeting intracellular antigens are generally considered diagnostic markers and are often associated with paraneoplastic neurological syndromes. In contrast, autoantibodies against extracellular antigens typically target specific extracellular domains, which usually exhibit direct pathogenic potential. These two classes of antibodies can coexist. Emerging evidence proposes a third subclassification, wherein specific intracellular antigens that are sometimes expressed on the cell surface are referred to as intracellular antigens with potential transient exposure on the plasma membrane [61]. Examples of this new category include amphiphysin and GAD65 associated with SPS [62, 63]. For the purposes of this review, these antigens are categorized as intracellular, associated conditions are included under intracellular antigen-mediated neurological autoimmune disorders treated with CAR T cell therapy. While our focus centers on autoimmune diseases driven by neural autoantibodies and their therapeutic implications, we do not elaborate on the relevance of paraneoplastic or idiopathic neurological syndromes [64]. Notably, specific neuronal and glial antigens contribute directly to pathophysiology and serve as important diagnostic biomarkers across neuroimmunological diseases, thereby informing potential therapeutic strategies. In this context, CAAR T cell therapy aims to leverage known pathogenic epitopes by selectively depleting autoreactive B cells that recognize these antigens, although its clinical applicability remains to be fully defined [47].

As the mechanisms of intracellular and extracellular autoantigens differ and the therapeutic principles of CAR T cells targeting intracellular antigen-mediated neurological disorders remain largely undisclosed, we categorized neurological autoimmune disorders into three groups: extracellular antigen-mediated disorders (e.g., MG, MOGAD, NMOSD, LEMS, and Anti-NMDAR encephalitis), intracellular antigen-mediated disorders (e.g., SPS, DAGLA antibody associated encephalitis), and disorders with incompletely defined or heterogeneous antigenic targets (e.g., MS). While candidate autoantigens such as myelin basic protein (MBP), proteolipid protein (PLP), and MOG have been proposed in MS, their roles remain inconsistent across studies; thus, MS is provisionally included in this category to highlight the ongoing uncertainty in its antigenic drivers [65, 66]. For each category, we detail known therapeutic targets, the current state of CAR T cell therapy in these diseases and propose potential mechanisms to develop a comprehensive understanding of the role of CAR T cell therapy in antigen-mediated neurological disorders (Table 3).

**Table 3:**
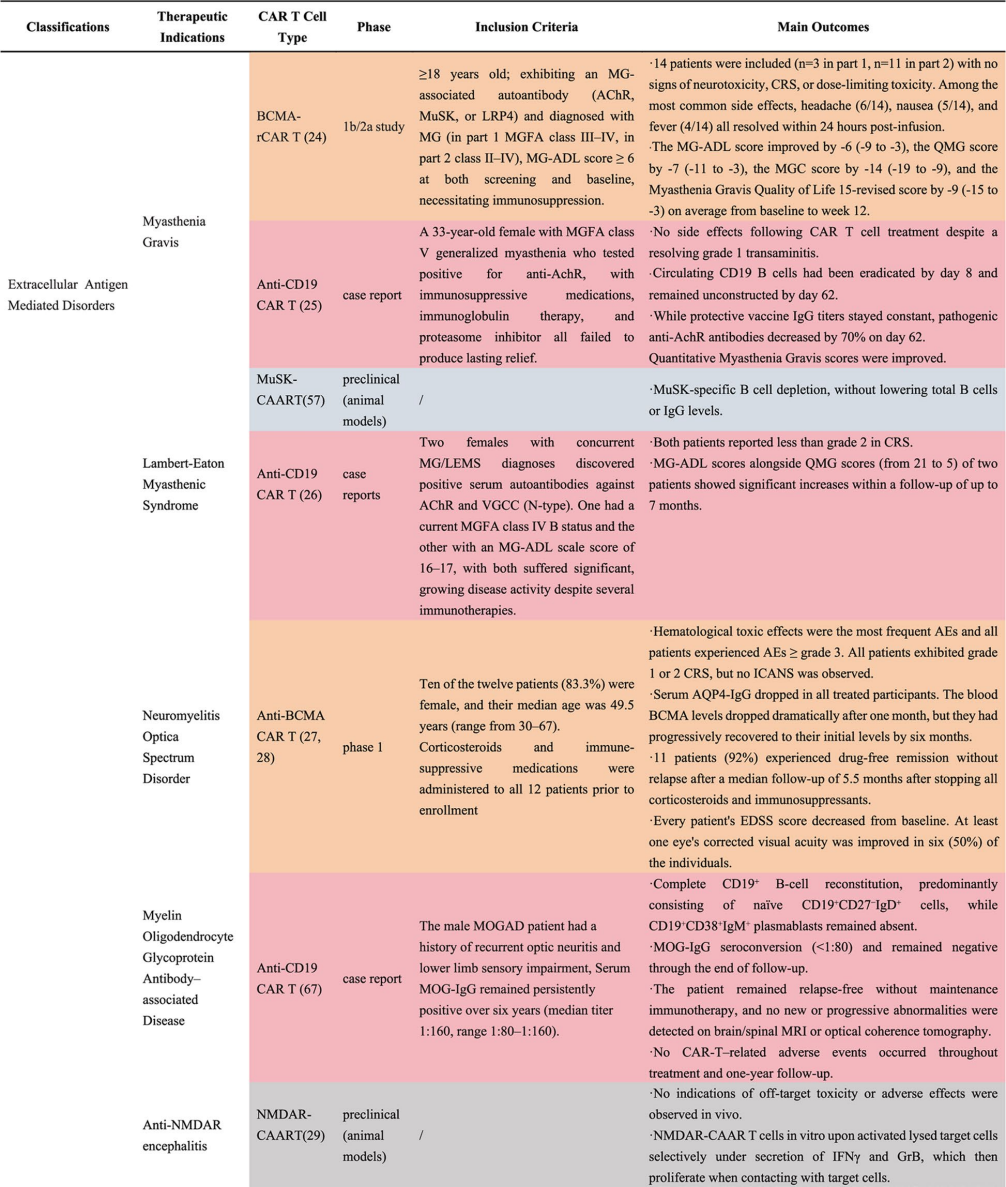

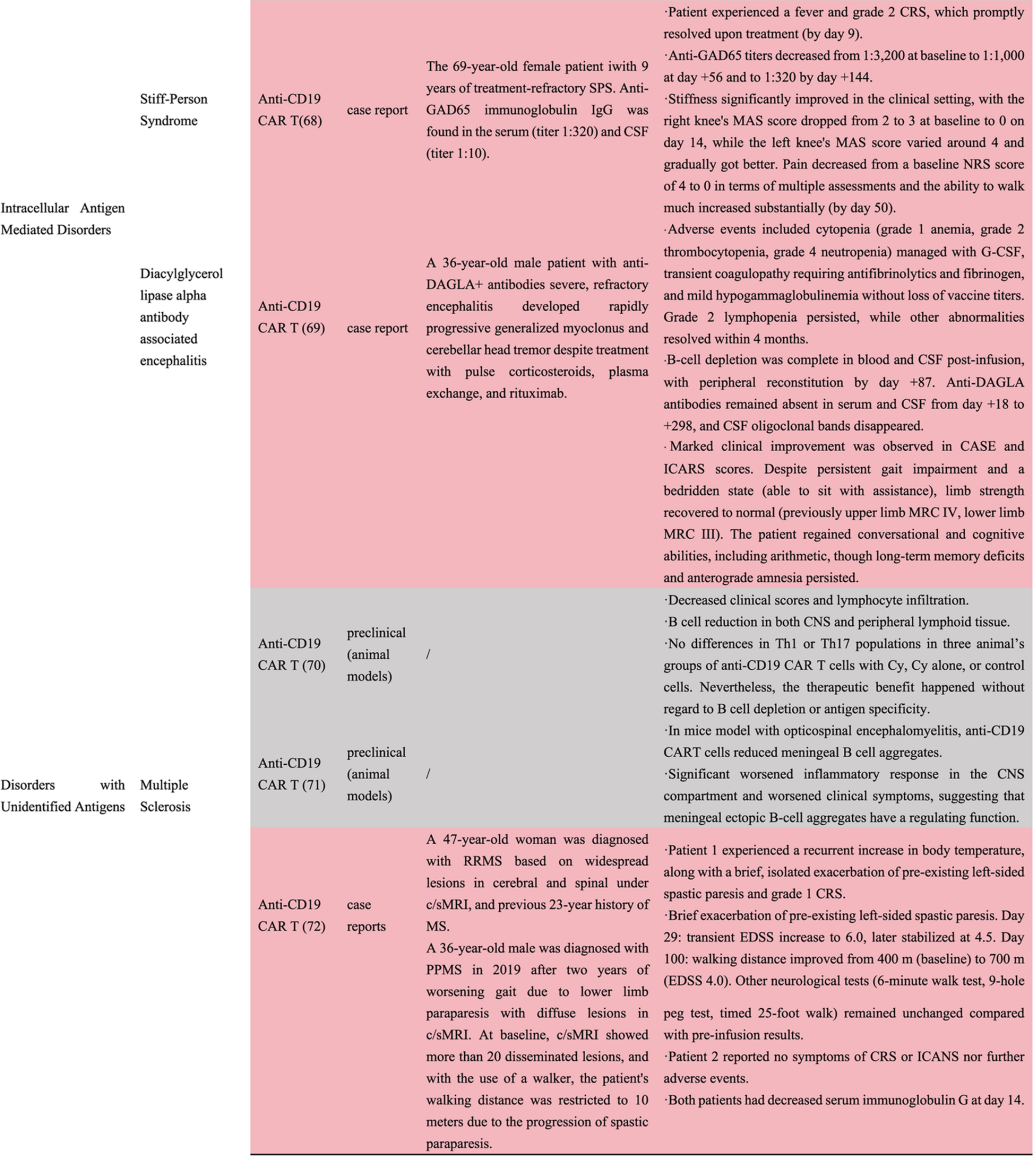
CAR T cell therapy in neurological autoimmune disorders

*Abbreviations: AChR* Anti-acetylcholine receptor, *c/sMRI* Cerebral and spinal magnetic resonance imaging, *CRS* Cytokine release syndrome, *QMG* Quantitative Myasthenia Gravis Score, *MGC* Myasthenia Gravis Composite, *MG-ADL* Myasthenia Gravis Activities of Daily Living Scale, *CSF* Cerebrospinal fluid, *EAMG* Experimental autoimmune myasthenia gravis, *EAE* Experimental autoimmune encephalomyelitis, *EDSS* Expanded disability status scale, *GrB* Granzyme B, *ICANS* Immune effector cell-associated neurotoxicity syndrome, *IFNγ *Interferon γ, *MAS* Modified Ashworth scale, *NRS* Numeric Rating Scale, *MG* Myasthenia gravis, *MG-ADL* Myasthenia gravis activities of daily living, *MGFA* Myasthenia Gravis Foundation of America, *NMDA* Anti-N-methyl-D-aspartate, *NRS* Numeric rating scale, *CASE* Clinical Assessment Scale in Autoimmune Encephalitis, *ICARS* International Cooperative Ataxia Rating Scale, *MRC* Medical Research Council, *PPMS* Primary-progressive Multiple sclerosis, *QMG* Quantitative myasthenia gravis, *RRMS* Relapsing-remitting Multiple sclerosis, *VGCC* Voltage-gated calcium channel

### CAR T cell therapy for autoimmune neurological disorders based on antigen classification

#### CAR T cell therapy for extracellular antigen-mediated neurological disorders

##### Myasthenia gravis

MG is a prototypical antibody-mediated neuromuscular autoimmune disorder in which pathogenic autoantibodies, most commonly targeting the AChR, lead to impaired neuromuscular transmission and manifest as fluctuating muscle weakness and fatigue [67, 68]. The severity of disease and treatment responses are clinically evaluated using standardized scales such as the Myasthenia Gravis Activities of Daily Living (MG-ADL) score, Quantitative Myasthenia Gravis (QMG) score, and Besinger disease activity score, where higher scores generally reflect greater functional impairment and disease burden. While antibodies against AChR, and less frequently MuSK or LRP4, provide valuable diagnostic and prognostic information, current therapies primarily modulate immune activity but do not adequately or specifically target the long-lived plasma cells responsible for sustained autoantibody production [69, 70]. Consequently, a subset of patients, particularly those with persistent AChR-positive antibodies, may show limited or incomplete responses to B cell depletion with rituximab [71, 72].

Even though new complement-targeting antibodies, such as eculizumab [40] and ravulizumab [73], have demonstrated effective treatment outcomes for highly active MG, their adoption is still accompanied by an incremental risk of infection. Additionally, up to 15% of patients show no response to complement therapy, likely due to C5 polymorphisms or alternative pathogenic mechanisms, such as antibody mediated AChR blockade or internalization. Neonatal Fc receptor (FcRn) inhibitors, which reduce circulating pathogenic antibody levels by disrupting IgG recycling and promoting its lysosomal degradation, have emerged as novel targeted immunotherapies in clinical practice. While FcRn antagonists have proven effective in alleviating autoantibody attacks on the neuromuscular junction (NMJ), these treatments still fail to eliminate harmful autoantibodies and avoid their downstream effects [74, 75]. Therefore, an urgent need for novel and more durable immunotherapies persists, with the selective expression of B cell maturation antigen (BCMA) on the surface of mature plasma cells and CD19 on B cells providing promising therapeutic opportunities. These considerations provide the rationale for exploring CAR T and CAAR T cell therapies in MG.

Cartesian Therapeutics reported a 12-month follow-up data from a Phase 2a clinical trial, an anti-BCMA autologous RNA chimeric antigen receptor T cell (rCAR T) therapy, for patients with refractory generalized MG. Among the 14 enrolled patients, significant and sustained clinical improvements were observed across four validated MG disease scoring systems, with an average improvement of 6 or more points in the MG-ADL score. Notably, no typical adverse effects related to CAR T cell therapy, such as neurotoxicity, CRS, hematologic abnormalities, functional immunosuppression, or opportunistic infections, were reported, demonstrating excellent immune tolerance. Clinically meaningful improvements in MG disease severity were accompanied by reductions in circulating plasma cell markers and trends indicating partial plasma cell depletion and slight decreases in total IgG, vaccine antibodies, and autoantibody concentrations [24].

Subsequently, a CD19-targeted CAR T therapy has been reported in a patient with severe refractory anti-AChR^+^ generalized MG. The patient showed substantial clinical improvement, evidenced by reductions in clinical multiparameter Besinger disease activity and QMG scores, correlated with serological elimination of CD19 B cells (cleared by day 8 and not reconstituted by day 62) and a marked decrease in pathogenic anti-AChR antibodies (from 2434 nmol/mL at day 0 to 718 nmol/mL at day 62). This study provided evidence that CD19^+^ plasmablasts together with short-lived plasma cells are responsible for producing most pathogenic anti-AChR autoantibodies. In contrast, protective autoantibodies are produced by long-lived CD19^-^ bone marrow plasma cells, unaffected by CD19 CAR T cells. The patient did not develop CRS, ICANS, hematopoietic insufficiency, or hypogammaglobulinemia but did experience self-limiting grade 1 transaminitis, which resolved without treatment [25].

For MuSK MG, MuSK-CAR T cells have also been specifically designed. In murine models, these cells exhibited efficacy comparable to CD19 CAR T therapy, with more stable retention times in the spleen. MuSK-CAR T cells specifically cleared anti-MuSK IgG without impacting overall IgG or B cell levels, thus circumventing the broad immunosuppressive limitations of current therapies. However, given the small sample sizes in these studies, the available evidence is limited in confirming the safety and efficacy of these therapies in MG patients, necessitating further experimental validation [57]. Nevertheless, the promising potential of CAR T therapy in achieving broad depletion of autoreactive cells and subsequent immune reset underscores its transformative role in neurological autoimmune diseases.

##### Lambert-Eaton myasthenic syndrome

LEMS is another neuromuscular autoimmune disease characterized by defective neurotransmitter release at the NMJ due to autoantibodies against presynaptic P/Q-type voltage-gated calcium channels (VGCCs) [76, 77]. Autonomic dysfunction, diminished deep tendon reflexes, and proximal muscular weakness are common triad symptoms [78]. For LEMS patients, although symptomatic therapies such as 3,4-diaminopyridine and immunosuppressive regimens provide benefit, these approaches do not specifically eliminate the pathogenic B cell and plasma cell populations that drive autoantibody production [79]. As a result, disease activity often persists, underscoring the need for more targeted and durable immunotherapies.

CD19-targeted CAR T therapy (KYV-101) has shown great efficacy, achieving both serological and clinical remission in two patients with severe, refractory disease characterized by the coexistence of myasthenia gravis and Lambert–Eaton myasthenic syndrome, in whom conventional therapies had either failed to halt progression or were discontinued due to adverse effects. Notable improvements were observed in patients’ quality of life and daily activities, as reflected in MG-ADL scores, particularly in mobility and gait. A significant decline in pathogenic autoantibodies accompanied these clinical improvements: anti-AChR autoantibodies decreased by 53% in patient 1 and completely normalized in patient 2, whereas anti-VGCC autoantibodies decreased by 96% in patient 1 and 93% in patient 2, subsequently returning to normal levels. Both patients remained disease-free and off treatment during the 7-month follow-up after data publication. Mild cytokine release syndrome occurred in both cases, and Patient 1 additionally developed Grade 1 ICANS, both of which resolved with treatment [26].

In the pathophysiology of LEMS, it has been established that autoantibodies mediate chronic autoimmune attacks, resulting in damage to the presynaptic membrane and reduced VGCC function. CD19-CAR T cell therapy not only achieved remarkable functional recovery but also led to complete immune reset in the cases with both conditions of long-standing MG as well as LEMS, underscoring its therapeutic potential for refractory autoimmune neuromuscular diseases. However, it remains unclear whether NMJ damage is reversible and to what extent it can recover [80].

##### Neuromyelitis optica spectrum disorder

NMOSD commonly presents with either monophasic or recurrent attacks of optic neuritis and transverse myelitis. It is a phenocopy that has been identified as distinct from MS since the presence of specific AQP-4-IgG autoantibody signature and the detection of consequent complement activation [81]. In most NMOSD cases, pathogenic autoantibodies AQP4-IgG are produced by plasma cells and mature B cells, which target AQP4 abundantly expressed on the optic nerve and spinal cord, leading to complement activation, astrocyte injury, and subsequent neuroinflammation [82]. Although several FDA-approved monoclonal antibodies, including anti-CD19 B cell–depleting antibody inebilizumab [11], IL-6R inhibitor satralizumab, C5 complement inhibitor eculizumab and ravulizumab [41] have substantially advanced NMOSD management, a proportion of patients continue to experience relapses despite current treatments [3, 83]. CAR T cell therapy, with its capacity to selectively and more thoroughly deplete autoreactive B cells thus eliminate pathogenic autoantibodies, offers a parallel yet potentially more durable approach, especially for patients with refractory symptoms.

Recently, a phase I pilot trial involving 12 adults with NMOSD treated with anti-BCMA CAR T cells showed that, over a 5.5-month follow-up period, 11 patients achieved drug-free remission without relapse. The decline in AQP4 antibody levels coincided with CAR T cell expansion, and improvements were generally reported in disability (Expanded Disability Status Scale (EDSS) scores), vision, walking ability, and bowel and bladder functions. All participants experienced grade 1–2 CRS and adverse events greater than grade 3, primarily manifesting as hematologic cytopenia and infections. Notably, when compared with the use of the exact same product of anti-BCMA CAR T cells in multiple myeloma, treatment in NMOSD patients was associated with significantly milder and shorter episodes of cytopenia, likely reflecting the absence of profound bone marrow involvement in this disease. The severity of CRS was also lower than typically reported in hematological settings, and infection rates were within the expected range for NMOSD patients receiving other immunotherapies. While established monoclonal antibody therapies have already provided substantial benefit and remain the current standard of care, these observations suggest that BCMA-CAR T cells may offer a comparatively favorable safety and tolerability profile in NMOSD, warranting further investigation [28].

Subsequently, the team conducted single-cell multi-omics analyses on both blood and CSF samples from AQP4^+^ NMOSD patients, revealing the evolution of CAR T cells *in vivo* at the cellular and molecular levels from a dynamic perspective. According to the study, CAR T cells with chemotactic characteristics can more readily cross the BBB, infiltrate the CNS, and directly eliminate autoreactive plasma cells, thereby reducing intrathecal autoantibody secretion and aberrant immune cell activation. At CSF immune microenvironment level, reductions were observed in pro-inflammatory cytokines, chemokines, and complement components, demonstrating that CAR T cells can regulate immune dysregulation of NMOSD patients especially within the CNS region. This reversal of immune dysfunction may underline the observed recovery following CAR T cell therapy. Thus, this discovery elucidates the crucial role of CAR T cells in correcting central immune abnormalities and reconstructing CNS immunity in patients, providing insights into potential applications for other autoimmune neurological disorders [27].

However, the adoption of CAR T cell therapy for NMOSD still remains in its early exploratory stage, likely attributed to its inherent complexities as a living drug and difficulties of recruiting less severe patients. In this context, it is important to acknowledge the considerably more mature clinical development of several monoclonal antibody therapies that have already achieved regulatory approval and demonstrated robust efficacy in NMOSD [11, 84–86].

#####  MOG antibody-associated disease

MOGAD is an autoimmune demyelinating disease characterized by perivenous demyelination, CD4⁺T cell infiltration and complement deposition in active lesions [87]. At present, due to the lack of high-quality clinical research evidence, the relapse prevention strategy of MOGAD and the treatment of refractory symptoms remains a challenge, while B cell depletion therapy is currently adopted in clinical practice, a substantial number of patients showed poor response to existing treatment [88].

A 25-year-old male patient with MOGAD had a long-standing history of recurrent optic neuritis and lower-limb sensory impairment, with an EDSS score of 7.5. Serum MOG-IgG remained persistently positive for six years (median titer 1:160). Following CD19-directed CAR T therapy, complete depletion of CD19⁺ B cells was achieved by day + 7 post-infusion, by day + 141, CD19⁺ B-cell reconstitution was noted, predominantly consisting of naïve CD19⁺CD27⁻IgD⁺ cells. During the one-year follow-up, CD19⁺CD27⁺IgD⁻ memory B cells gradually reappeared, while CD19⁺CD38⁺IgM⁺ plasmablasts remained persistently low (< 0.5%) or absent. MOG-IgG seroconversion occurred on day + 22 (< 1:80) and remained negative through the end of follow-up. Notably, an optic neuritis relapse developed on day 29, despite persistently negative MOG-IgG status. The patient remained relapse-free afterwards without maintenance immunotherapy, and no new or progressive abnormalities were detected on brain/spinal MRI or optical coherence tomography. No CAR T–related adverse events occurred throughout treatment and follow-up [89]. This single-case observational study demonstrates that CD19-targeted CAR T therapy is well tolerated in refractory MOGAD. The sustained MOG-IgG negativity paralleled the depletion of memory B cells and plasmablasts, suggesting that MOG-IgG is primarily derived from CD19⁺ memory B cells and plasmablasts, while long-lived CD19⁻ plasma cells maintain immune tolerance. These findings warrant confirmation in multicenter, large-scale studies.

##### Anti-NMDAR encephalitis

Anti-NMDAR encephalitis is currently the most prevalent type of AE. It is associated with autoantibodies targeting the NR1 subunit of NMDAR, an extracellular ionotropic glutamate receptor located on the neuronal cell surface. One crucial diagnostic criterion is the existence of IgG NMDAR antibodies in CSF, and the functions of B cells and plasma cells in disease progression have been well established. However, a subset of patients still experiences severe neurological sequelae despite receiving intensive immunosuppressive therapy [90].

Therefore, Reincke et al. proposed a more specific treatment to selectively deplete NMDAR autoantibody-producing B cells. They developed NMDAR-specific chimeric autoantibody receptor (NMDAR-CAAR) T cells, comprised of a 4-1BB/CD3 intracellular signaling domain fused to a fragment of the NMDAR autoantigen. *In vitro*, the CAAR T cells bound to various NMDAR autoantibodies generated from human patients and conducted effective target cell lysis. Effector molecules were secreted after being activated upon target cell encounter, then proliferation and specific killing took place. Notably, the NMDAR-CAAR T cells did not affect healthy donors’ primary B cells, meanwhile, a decrease in brain and serum autoantibodies was seen in a mouse model, indicating that CAR T cells successfully and precisely depleted pathogenic B cells expressing antibodies that are NMDAR-reactive, with no symptoms of adverse events or off-target toxicity [29]. However, this result is limited using a passive transfer B cell model that does not reproduce the full neuropathological or behavioral phenotype of Anti-NMDAR encephalitis, as well as by the restricted antibody diversity analyzed, necessitating future studies employing active immunization and broader patient-derived antibody panels.

In addition to its application in Anti-NMDAR encephalitis, preclinical studies for MuSK-MG, PV, and type 1 diabetes (T1D) have explored the adoption of CAAR T in autoimmune diseases. Subsequent sections discuss these studies in greater detail [57, 91–93].

#### CAR T cell therapy for intracellular antigen-mediated neurological disorders

##### Stiff-Person syndrome

SPS is a prototypical autoimmune neuronal hyperexcitability condition associated with impaired GABAergic synaptic transmission. Autoantibodies produced by the immune system primarily target presynaptic GAD-65, amphiphysin, and postsynaptic GABA receptor-associated proteins, such as gephyrin. The recently proposed diagnostic criteria for SPS emphasize: (1) the presence of anti-GAD65 antibodies in serum and/or CSF (2), the presence of anti-GlyR antibodies in serum and/or CSF, or (3) the presence of anti-amphiphysin antibodies in serum and/or CSF. High-titer autoantibodies that target linear epitopes of GAD65 were found to correlate to the severity of the illness, indicating a strong T cell response to GAD65 [94], while anti-GlyR antibodies directly impair inhibitory neuronal signaling, and amphiphysin antibodies reduce presynaptic GABA release, both leading to neuronal hyperexcitability [95]. This underscored the importance of autoantibodies not only for diagnosis but also as evidence of their pathogenic contribution. Thus, eliminating pathogenic antibodies theoretically alleviates disease progression [96–98]. However, intracellular antigens are theoretically inaccessible to circulating antibodies, and the prevailing view is that antibodies targeting intracellular proteins such as GAD65 primarily serve as disease markers rather than direct pathogenic mediators [99, 100]. Consequently, their pathogenic potential remains controversial. This discrepancy emphasizes the need for further studies to clarify their precise role in disease pathogenesis, to determine whether eliminating such antibodies can provide meaningful clinical benefit, and to elucidate the underlying mechanisms.

A female patient with a 9-year history of refractory SPS received CD19 CAR T cells (KYV101). The anti-GAD65 titer decreased from 1:3200 at baseline to 1:1000 at day + 56, then to 1:320 five months post-treatment. This was accompanied by significant clinical improvements in stiffness, pain relief and walking ability. Transient fever occurred, which resolved after treatment. Importantly, consistent with the experience of employing anti-CD19 CAR T cells for autoimmune disorders such as MG, MS, SLE, and RA, no severe safety signals (ICANS or CRS greater than grade 3) were observed in CAR T clinical outcomes. Severe neurotoxicity events were also substantially lower than those reported in B cell lymphoma, suggesting a potentially acceptable safety profile [101]. However, as this report represents only a single case, caution is required in drawing generalizable conclusions, and additional evidence is warranted. In this context, the ongoing clinical trial of CD19-CAR T therapy in SPS (NCT06588491) will provide more data.

##### Diacylglycerol lipase alpha (DAGLA) antibody associated encephalitis

DAGLA antibody associated encephalitis is a novel type of autoimmune cerebellitis, characterized by autoantibodies against DAGLA being detected in CSF and serum samples [102]. DAGLA localizes as a multipass membrane protein expressed on the surface of the postsynaptic dendritic spines of hippocampus neurons and cerebellar Purkinje cells, where it regulates endocannabinoid signaling through the generation of 2-arachidonoylglycerol (2-AG) from the cytoplasmic side of the plasma membrane. It is indicated by epitope analyses that autoantibodies predominantly recognize intracellular linear epitopes [103, 104]. The response to immunotherapy in previous study of four patients was limited, with only moderate or short-lived improvement observed. Initial corticosteroid treatment had minimal impact on the progression of clinical symptoms, and all patients exhibited persistent severe neurological deficits. Improvements following immunoadsorption, plasma exchange, and IVIG therapy were transient. Therefore, early diagnosis, along with more intensive and prolonged immunotherapy, may yield greater benefits and potentially prevent irreversible cerebellar damage [102].

In a recently published study in July 2025, CD19-directed CAR T-cell therapy successfully treated a case of refractory anti-DAGLA antibody-associated autoimmune encephalitis [105]. A 36-year-old patient, who had failed to respond to conventional therapies including corticosteroids, plasma exchange, and rituximab, received a single infusion of autologous anti-CD19 CAR T cells. Following treatment, the patient demonstrated marked clinical improvement, as assessed by the Clinical Assessment Scale in Autoimmune Encephalitis (CASE) and the International Cooperative Ataxia Rating Scale (ICARS). Levels of anti-DAGLA antibodies decreased in both serum and CSF. Complete depletion of B cells was observed in peripheral blood and CSF, accompanied by the elimination of anti-DAGLA antibodies in both compartments. It is of significant value to investigate whether anti-DAGLA autoantibodies exert direct pathogenic effects, potentially by inhibiting or activating protein function, triggering complement-mediated cytotoxicity, or promoting T cell–mediated processes. Further studies are needed to explore these mechanisms across different patient cohorts and to elucidate how DAGLA antibodies may affect neuronal function. For antibody-mediated CNS autoimmune diseases, this case offers early evidence that severe B-cell depletion with CD19-CAR T cell therapy may be a unique and effective therapeutic approach, especially in instances that are resistant to previous treatment.

#### CAR T cell therapy for neurological autoimmune disorders without identified antigen

##### Multiple sclerosis

MS is a chronic demyelinating disease of the CNS with no known specific cause, characterized by a diverse range of symptoms and signs due to varying involvement of autonomic, sensory, motor, and visual systems. The infiltration of immune cells into the CNS is a hallmark of MS, but traditional therapies have limited effects on these tissue-resident immune cells [106]. To date, no specific autoantibody has been confirmed as pathogenic or pathognomonic, and disease progression is thought to be driven instead by compartmentalized inflammation, microglial activation, and neurodegenerative processes [6]. This immunopathological complexity has made prevention of disease progression particularly challenging despite advances in disease-modifying therapies. Nevertheless, emerging research has provided deeper insights into the complex autoimmune and neurodegenerative mechanisms underlying MS, with particular emphasis on the pivotal role of B cells in disease pathophysiology and relapse reduction. B cells produce autoreactive antibodies and activate autoreactive T cells by presenting self-reactive peptides via MHC [6]. Although no therapy can completely prevent or reverse the progressive neurodegeneration, immune reconstitution therapy has demonstrated potential in inducing MS patients into long-term or possibly permanent drug-free remission [107–109], with selective B cell depletion therapies—such as rituximab, ocrelizumab, and ofatumumab, show promise in effectively reducing inflammation originating in the periphery, highlighting the crucial role of B cells in MS pathology [44, 110, 111]. However, growing evidence suggests that relapse prevention does not necessarily equate to control over disease progression [30, 45].

The persistence of oligoclonal bands (OCBs) in CSF despite peripheral immunosuppression alongside B cell depletion in the CSF suggests that CNS-compartmentalized, tissue-resident B lymphocytes (including CD20⁻ long-lived plasma cells) may not be effectively eliminated. This observation is supported by studies showing ongoing intrathecal clonal B-cell / plasma-cell populations and persistent CSF OCBs following anti-CD20 therapy [112, 113]. Therefore, using CAR T cells, which have shown the capacity to enter deeper tissue compartments, to eliminate pathogenic B cells may potentially alter the clinical outcomes of monoclonal antibody therapies, achieving more comprehensive immune reconstitution [30]. In B cell-dependent experimental autoimmune encephalomyelitis (EAE) mouse models, CD19 CAR T cell therapy improved EAE symptoms and delayed disease onset [114]. However, contradictory results were observed in another mouse model, where CD19 CAR T administration exacerbated EAE within two weeks, accompanied by widespread demyelination and axonal loss [115]. Observational evidence, however, has started to offer more realistic insights into CAR T cell therapy’s potential. In a recent case series, CAR T cell treatment demonstrated significant benefits in two progressive MS patients, resulting in the enrichment of CAR T cells in CSF along with reduced intrathecal antibody levels with no early signs of neurotoxicity, which underscores the advantages of CAR T cell therapy in penetrating immune-privileged compartments. The study also highlights the therapeutic potential of CD19 CAR T cells for advanced MS patients unresponsive to traditional B cell depletion therapies, as they more thoroughly eradicate CNS-resident B lymphocytes that responsible for accelerating the course of the disease and inflammatory relapses [116]. Notably, several registered trials (e.g. NCT06384976, NCT06451159, NCT07178431, NCT06138132, NCT06220201) are currently underway, which is expected to provide further supportive evidence to determine this therapy’s short- and long-term safety and efficacy of CAR-T cell therapy in patients with refractory or progressive MS.

**Fig. 2:**
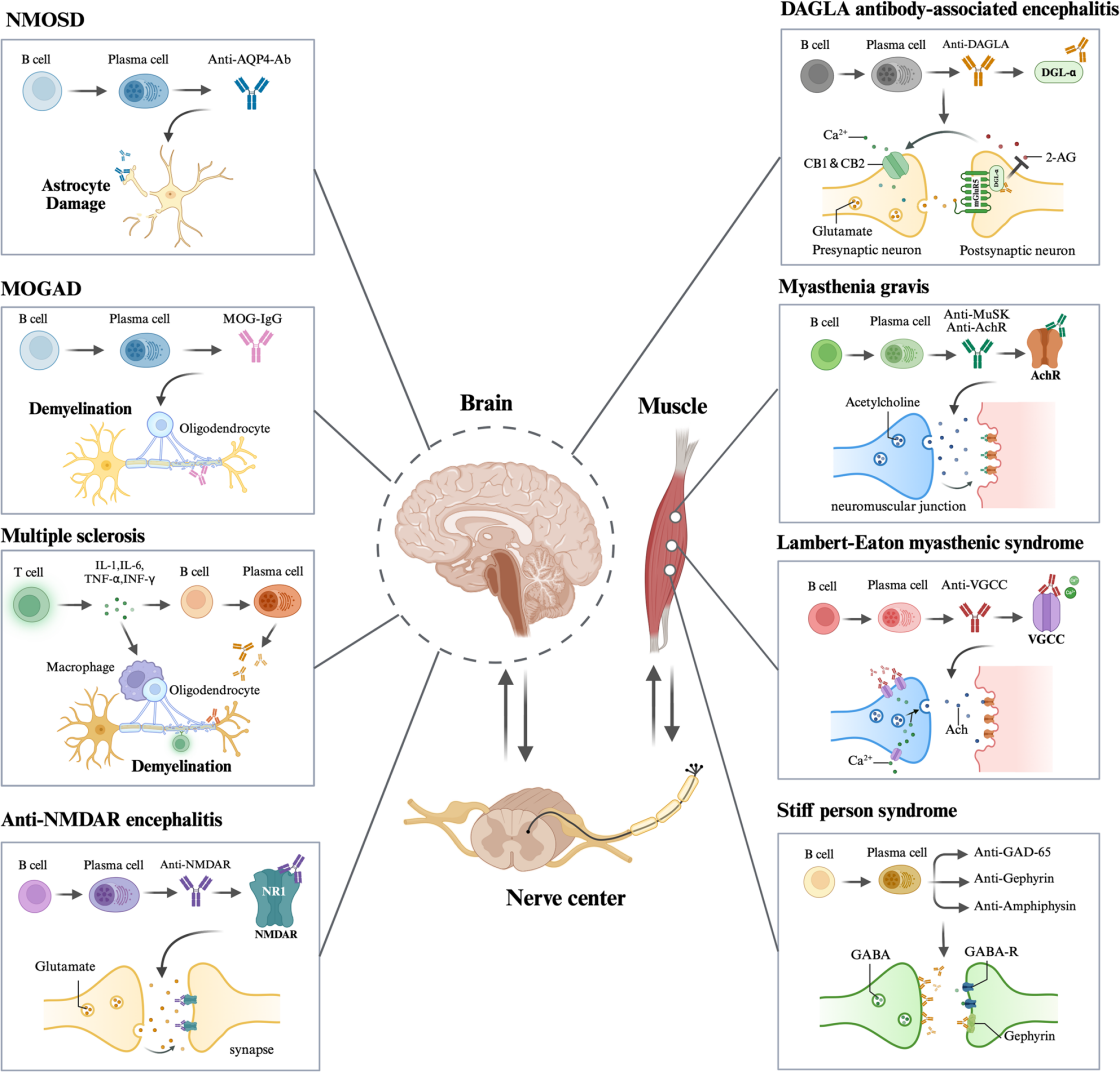
Roles of aberrant B cells and autoantibody in the pathophysiology in autoimmune neurological disorders The pathogenic targets and mechanisms related to implicated autoantibodies of eight autoimmune disorders (NMOSD, MOGAD, MS, NMDAR encephalitis, DAGLA antibody-associated encephalitis, MG, LEMS, SPS) is summarized. NMOSD is mediated by AQP4 antibodies, leading to astrocyte damage. MOGAD, demyelinating disease induced by MOG-IgG binding to oligodendrocytes. MS involves T cell and B cell activation, releasing proinflammatory cytokines (IL-1, IL-6, TNF-α, IFN-γ) and inducing oligodendrocyte damage, resulting in demyelination. Anti-NMDAR encephalitis is characterized by autoantibodies targeting NMDAR at the synapse, disrupting glutamatergic neurotransmission. DAGLA antibody-associated encephalitis is caused by anti-DAGLA autoantibodies disrupting 2-AG–mediated endocannabinoid signaling at excitatory synapses. MG is caused by autoantibodies against AChR or MuSK at the neuromuscular junction, impairing synaptic transmission. LEMS is mediated by VGCC antibodies, reducing calcium influx and acetylcholine release. SPS is associated with antibodies against GAD65, gephyrin, and amphiphysin, affecting GABAergic inhibitory signaling. These autoimmune disorders underscore the critical role of B cell-mediated humoral immunity in neuroimmunological diseases and better our understanding for the principle of CAR T cell therapy upon these disorders

It is worth mentioning that CAR Treg therapy has shown particularly promising results in MS. While Tregs naturally provide protective effects in EAE, their differentiation into Th17 cells in the absence of TGF-β contributes to MS pathology. To address this, Moa Fransson et al. engineered CARs expressing FoxP3 while targeting MOG in CD4^+^ T cells (CARαMOG-FoxP3). FoxP3 expression drives the Treg phenotype via transcriptional regulation, while CARαMOG promotes Treg binding to MOG-expressing oligodendrocytes [117]. The engineered Tregs efficiently penetrated brain regions following intranasal administration, suppressing EAE progression and reducing disease symptoms. In treated mice, improved myelination, reduced reactive astrogliosis, and increased levels of myelin basic protein (MBP) levels and GFAP confirmed CAR Treg efficacy. Additionally, brain tissues showed lower mRNA levels of IFN-γ and IL-12 [118]. The therapy of autoimmune diseases may benefit greatly from both CAR Treg and CAAR T treatments, as they provide antigen-specific targeting while minimizing systemic immunosuppression. Nonetheless, future studies must overcome challenges such as identifying optimal CAR targets, reducing costs, and developing scalable approaches for broader clinical applications. In this section, potential mechanisms regarding CAR T therapy in diseases mentioned above are shown in Fig. 2.

### CAAR T cell and CAR-Treg therapy for wider autoimmune diseases

Beyond autoimmune disorders directly linked to the nervous system [119, 120], researchers are also focusing on other autoimmune conditions, including SLE, rheumatoid arthritis (RA), SSC, IIM, anti-synthetase syndrome, PV, Crohn’s disease, and T1D. Among these, the application of CAR T cells has been extensively explored, with increasing data from follow-ups being summarized and presented [20, 21, 91, 121–125]. Notably, various CAR T cell types are being adopted, including derivative approaches such as CAAR T and CAR Treg therapies, which have shown promising outcomes across multiple autoimmune diseases.

CAAR T cell therapy demonstrates superior specificity by selectively eliminating B cells that produce anti-autoantigen antibodies, which may theoretically lead to milder immunosuppressive side effects due to its limited clearance of components. Beyond NMDA receptor encephalitis and MuSK-MG discussed above, preclinical studies of CAAR T-cell therapy have also been conducted in PV and type 1 diabetes. In PV, where autoantibodies target desmoglein 3 (DSG3), DSG3-CAAR T cells were reported to have nearly eradicated all DSG3-specific B cells in both *in vivo and in vitro* mouse models, significantly reducing anti-DSG3 antibody levels together with novel symptoms improvements without evident toxicity, while preserving other B cell populations [91]. In terms of T1D, CAAR T cell therapy targeting I-Ag7-B:9–23(R3) was developed yet disappointing results indicated only week-long disease onset delay and failed to prevent disease progression [92], whereas InsB: R3-CAR Tregs effectively suppressed autoimmunity and demonstrated significant prevention of T1D progression [93]. In summary, these contrasting outcomes highlight the importance of precise epitope selection in CAAR T design, and that autoimmune diseases with well-defined epitopes could be effectively targeted through CAAR T or CAR-Treg therapies within correct recognition of pathogenic autoantibodies and design [47]. Moreover, therapeutic efficacy can serve as reverse evidence supporting the pathogenicity of disease-specific epitopes, a concept that may be extended to other neuroimmunological disorders, especially those with unclear pathological procedure.

On the other hand, Tregs, a CD4 + T cell-subset that regulates immune tolerance, was proven to be attractive tools for managing autoimmune disorders [126, 127], especially when under redirection to focus on antigen-expressing targets, thus lowering the possibility of systemic immunosuppression. CAR Tregs have expanded application to animal models of T1D, MS, and vitiligo, based on preclinical and clinical results [118, 125, 128, 129]. Tenspolde et al. first generated FOXP3-engineered insulin-specific CAR Tregs, but they failed to prevent diabetes in NOD mice [125]. Similarly, targeting the pancreatic marker HPi2 was unsuccessful due to its broad CD4⁺ T-cell expression, causing uncontrolled proliferation [130]. In contrast, GAD65-CAR Tregs in humanized T1D mice significantly improved glucose tolerance, along with β cell recovery and regeneration [131]. These findings highlighted the importance of precise antigen selection of optimal CAR targets.

## Conclusion

Autoantibodies are produced by plasmablasts, short-lived plasma cells, and long-lived plasma cells. Targeting these cells offers the potential to eliminate the sources of autoantibody production, serving as a promising strategy for the treatment of B cell-mediated autoimmune diseases. CAR T cell therapy has demonstrated the ability to achieve thorough clearance of pathogenic target cells within tissues, effectively creating a “new” immune system, with the reconstituted system seemingly lacking the same predisposition to generate pathogenic B cells, thereby offering the prospect of a complete immune system reset. This could lead to long-term drug-free remission or even a complete cure for autoimmune diseases; however, extended follow-up durations and larger clinical samples are required to substantiate these findings.

Based on the antigenic sites of autoimmune neurological diseases, extracellular antigen-mediated conditions, such as MG, NMOSD, and Anti-NMDAR encephalitis, could potentially benefit from CAR T cell therapy under similar principles [132]. However, for neurological autoimmune disorders with intracellular antigenic sites, whose pathogenic mechanisms remain unclear, the limited development of targeted therapies highlights the significance of CAR T cell therapy as an emerging treatment, facilitating both therapeutic exploration and mechanistic research. The same principle applies to diseases like MS, where the pathogenic antigen has yet to be clearly identified. As seen in SLE, the role of aberrant B cell activation in SLE has been further elucidated based on the outcomes gained between the adoption of monoclonal antibodies and CAR T cell therapies, also revealing the concept of autoantibody production by distinct cell populations (short-lived versus long-lived plasma cells) with varying sensitivities to B cell depletion therapies [33]. Anti-dsDNA antibodies, for instance, are hypothesized to originate preferentially from CD19 ^+^ plasmablasts or short-lived plasma cells rather than an entire group of aberrant B cells. Similar mechanistic analyses can be applied to autoimmune neurological diseases. In MG, for example, autoantibodies appear to arise primarily from plasmablasts or short-lived plasma cells, as targeting CD19 and BCMA with CAR T cell therapies has shown favorable efficacy, while rituximab, which targets CD20 has also demonstrated to additionally decrease AChR autoantibody levels [33, 133].

At the same time, target selection in CAR T cell therapy represents both an opportunity and a challenge. Especially in neurological autoimmune diseases where the pathogenic antigen is either intracellular or not clearly identified—as categorized in our review—evaluating the therapeutic responses of CAR T cells targeting different epitopes may help elucidate the actual disease-driving mechanisms. This approach may be particularly valuable for dissecting the pathogenic basis of neuroimmune autoimmune disorders and guiding the rational design of targeted immunotherapies in the future.

While any autoimmune diseases responsive to B cell depletion therapies could be considered as a potential candidate for CAR T cell therapy, differences in disease context must be acknowledged. Compared to relapsed end-stage cancer patients, individuals with autoimmune diseases tend to be younger, healthier, and exhibit significantly lower mortality rates. Therefore, for autoimmune patients in better health status with much lower mortality rates, it might not be worth it for them to undertake the risk of severe side effects from CAR T cell therapy. This reveals another challenge for further public adoption of CAR T cell therapy on autoimmune disease regardless of its previous outcomes gained, that a delicate balance between the cost of immunosuppression and the treatment efficacy must be carefully evaluated: the risk of excessively eliminating immune cells might lead to long-term immunodeficiency and more side effects. Therefore, CAR T cell therapy candidates are more likely to be patients with dominant B cell pathology and life-threatening disease severity who have failed to respond to safer and more effective treatments. Regarding CAR T cell therapy-related adverse effects, CRS, which occurs in 40% to 80% of hematologic malignancy patients receiving CAR T cell therapy, appears to be rarer and milder in autoimmune diseases [134]. Schett and his colleagues hypothesized that the lower risk of CRS may result from the lower circulating B cell counts in these patients compared to cancer patients, with the lower B cell burden in autoimmune diseases suggesting a better immune tolerance profile [135].

Building on the currently registered clinical trials summarized in Table 4, several practical limitations must still be addressed before broader clinical translation can be realized. Recruitment remains a major hurdle, as identifying patients who are sufficiently treatment-refractory yet stable enough to tolerate a living-drug therapy requires careful calibration of inclusion criteria. Moreover, optimal dosing regimens, lymphodepletion strategies, and monitoring frameworks have not been standardized, leaving uncertainty about how to maximize therapeutic benefit while minimizing prolonged immunosuppression. Equally, long-term risks and durability of response require systematic evaluation and validation in larger and more diverse cohorts. These considerations underscore that, despite promising early signals, the field must continue refining trial design and safety assessment to support the responsible expansion of CAR T cell therapy in neurological autoimmune diseases.

**Table 4:** Ongoing and registered clinical trials investigating CAR T cell therapy in neurological autoimmune diseases

Disease	Phase	Trial (Official Title)	Intervention	CAR T Target	NCT number	Status
Myasthenia Gravis (MG)	Phase IIb	Autologous T-Cells Expressing A Chimeric Antigen Receptor Directed To BCMA In Patients With Generalized MG	Descartes-08	BCMA	NCT04146051	Active / not recruiting
	Phase II/III	KYSA-6: A Phase 2/3, Open-Label, Randomized, Controlled, Multicenter Study of KYV-101, an Autologous Fully Human Anti CD19 CAR T Therapy, Versus Ongoing Standard-Of-Care Immunosuppressive Therapy in Patients with Generalized MG	KYV-101	CD19	NCT06193889	Recruiting
	Phase I	Evaluate the Safety and Efficacy of CD19-BCMA Targeted CAR T Therapy for Refractory, Generalized MG: A Single-center, Open-label, Single-arm, Dose-finding Study	CD19-BCMA Targeted CAR T	CD19-BCMA	NCT06371040	Recruiting
Stiff Person Syndrome (SPS)	Phase II	KYSA-8: A Phase 2 Open-Label, Single-Arm, Multicenter Study of KYV 101, an Autologous Fully Human Anti-CD19 CAR T Therapy, in Subjects with Treatment Refractory SPS	KYV-101	CD19	NCT06588491	Recruiting
Multiple sclerosis (MS)	Phase II	KYSA-7: A Phase 2, Open-Label, Randomized, Multicenter Study of KYV-101, an Autologous Fully Human Anti-CD19 CAR T Therapy, in Subjects with Refractory Primary and Secondary Progressive MS	KYV-101	CD19	NCT06384976	Active / not recruiting
	Phase I	Phase 1, Open-Label, Single Center Study of KYV-101, an Autologous Fully Human Anti-CD19 CAR T Therapy, in Participants with Treatment Refractory Progressive MS	KYV-101	CD19	NCT06451159	Active / not recruiting
	Phase I/II	An Open Label Phase I/IIa, Multicenter, Interventional Single-arm Trial of MB-CART 2019.1 in Patients with Refractory MS	MB-CART2019.1 (industry CAR)	/	NCT07178431	Not yet recruiting
Relapsing Forms of Multiple Sclerosis (RMS), Progressive Forms of Multiple Sclerosis (PMS), or Refractory MG.	Phase I	A Phase 1, Multicenter, Single-arm, Dose-escalation Study of CC-97,540 (BMS-986353), CD19-Targeted NEX-T CAR T Cells, Evaluating Safety and Tolerability in Participants with Autoimmune Neurological Diseases: Relapsing Forms of RMS, PMS, or Refractory MG.	CC-97,540 (BMS-986353)	CD19	NCT06220201	Recruiting
NMOSD, MG, MS, Chronic Inflammatory Demyelinating Polyradiculoneuropathy (CIDP), Autoimmune Encephalitis	Phase I	A Clinical Study Evaluating the Safety and Preliminary Efficacy of Universal Allogeneic CAR T-cell Therapy Targeting CD19 and BCMA in Patients with Relapsed/ Refractory Neurological Autoimmune Diseases	Universal CD19/BCMA CAR T	CD19/BCMA	NCT06939166	Not yet recruiting
NMOSD, MG, CIDP, IIM, MS, autoimmune encephalitis, MOGAD and POEMS Syndrome.	Early Phase I	An Open Label Clinical Trial to Evaluate the Safety and Efficacy of CT103A Cells for the Treatment of Relapsed/ Refractory Antibody-associated Inflammatory Diseases of the Nervous System	CT103A	BCMA	NCT04561557	Recruiting
MS, NMOSD, MOGAD, MG	Phase I	An Open Clinical Study to Evaluate the Safety, Tolerability, Pharmacokinetics and Pharmacodynamics of LCAR-AIO CAR-T Cells for Treating Relapsed/Refractory Neurological Autoimmune Diseases	LCAR-AIO	/	NCT06869278	Recruiting

We searched ClinicalTrials.gov for registered CAR T clinical trials in neurological autoimmune diseases. Table 4 lists the ongoing and registered trials we identified, along with NCT identifiers and sources. For several disease entities (LEMS, Anti-NMDAR encephalitis, DAGLA encephalitis) no dedicated CAR T registered trials were found at the time of search, indicating evidence gaps.

In summary, longer experimental durations, larger sample sizes, and rigorous long-term safety monitoring are crucial for advancing the application and maturation of CAR T cell therapy in autoimmune neurological diseases. Despite current challenges, CAR T cell therapy represents a highly promising and revolutionary approach with significant potential to improve patient outcomes.

## Data Availability

No datasets were generated or analysed during the current study.

## Abbreviations

AChR: Acetylcholine Receptor
ADEM: Acute Disseminated Encephalomyelitis
AGNA: Antiglial Nuclear Antibody
AK5: Adenylate Kinase 5
AMPAR: α-amino-3-hydroxy-5-methyl-4-isoxazolepropionic Acid Receptor
ANNA: Antineuronal Nuclear Antibody
APCs: Antigen-Presenting Cells
AQP4: Aquaporin-4
BBB: Blood–Brain Barrier
BCMA: B cell Maturation Antigen
CAAR: Chimeric Autoantigen Receptor
CAPD score: Cornell Assessment of Pediatric Delirium score
CAR: Chimeric Antigen Receptor
CASE: Clinical Assessment Scale in Autoimmune Encephalitis
CASPR2: Contactin-Associated Protein-Like 2
CNS: Central Nervous System
CRS: Cytokine Release Syndrome
CSF: Cerebrospinal Fluid
c/sMRI: Cerebral and Spinal Magnetic Resonance Imaging
DAGLA: Diacylglycerol Lipase Alpha
DSG 3: Desmoglein 3
EAMG: Experimental Autoimmune Myasthenia Gravis
EAE: Experimental Autoimmune Encephalomyelitis
EDSS: Expanded Disability Status Scale
FcRn inhibitors: Fc Receptor Neonatal Inhibitors
GABA: γ-aminobutyric Acid
GAD65: Glutamic Acid Decarboxylase 65
GFAP: Glial Fibrillary Acidic Protein
GluR1/2: Glutamate Receptor 1/2
GrB: Granzyme B
ICANS: Immune Effector Cell-Associated Neurotoxicity Syndrome
ICE score: Immune Effector Cell-Associated Encephalopathy score
ICARS: International Cooperative Ataxia Rating Scale
IFN: Interferon
IIM: Idiopathic Inflammatory Myopathies
IVIG: Intravenous Immunoglobulin
LE: Iimbic Encephalitis
LEMS: Lambert-Eaton Myasthenic Syndrome
LGI1: Leucine-Rich Glioma-Inactivated 1
LLN: Lower Limit of Normal
MAS: Modified Ashworth Scale
MBP: Myelin Basic Protein
MG: Myasthenia Gravis
MG-ADL: Myasthenia Gravis Activities of Daily Living
MGFA: Myasthenia Gravis Foundation of America
MHC: Major Histocompatibility Complex
mGluR5: Metabotropic Glutamate Receptor 5
MOG: Myelin Oligodendrocyte Glycoprotein
MOGAD: Myelin Oligodendrocyte Glycoprotein Antibody-Associated Disease
MRC: Medical Research Council
MS: Multiple Sclerosis
MuSK: Muscle-Specific Tyrosine Kinase
NMDAR: N-Methyl-D-Aspartate Receptor
NMJ: Neuromuscular Junction
NMOSD: Neuromyelitis Optica Spectrum Disorder
NR1: N-Methyl-D-Aspartate Receptor Subunit 1
NRS: Numeric Rating Scale / numeric rating scale
OCBs: Oligoclonal Bands
PCA1/2: Purkinje Cell Antigen 1/2
PLP: Proteolipid Protein
PPMS: Primary-Progressive Multiple Sclerosis
PV: Pemphigus Vulgaris
QMG: Quantitative Myasthenia Gravis
RA: Rheumatoid Arthritis
RRMS: Relapsing-Remitting Multiple Sclerosis
scFv: single-chain Fragment variables
SLE: Systemic Lupus Erythematosus
SPS: Stiff Person Syndrome
SS: Sjögren’s Syndrome
TAAs: Tumor-Associated Antigens
T1D: Type 1 Diabetes
TCR: T cell Receptor
TNF: Tumor Necrosis Factor
Tregs: Regulatory T cells
VGCC: Voltage Gated Calcium Channel
VGKC: Voltage-Gated Potassium Channels
2-AG: 2-arachidonoylglycerol
